# Extracellular Vesicles from Human Teeth Stem Cells Trigger ATP Release and Promote Migration of Human Microglia through P2X4 Receptor/MFG-E8-Dependent Mechanisms

**DOI:** 10.3390/ijms222010970

**Published:** 2021-10-11

**Authors:** Ugnė Jonavičė, Diana Romenskaja, Karolina Kriaučiūnaitė, Akvilė Jarmalavičiūtė, Justina Pajarskienė, Vytautas Kašėta, Virginijus Tunaitis, Tarja Malm, Rashid Giniatulin, Augustas Pivoriūnas

**Affiliations:** 1 Department of Stem Cell Biology, State Research Institute Centre for Innovative Medicine, LT-01102 Vilnius, Lithuania; ugne.jonavice@imcentras.lt (U.J.); diana.romenskaja@imcentras.lt (D.R.); karolina.kriauciunaite@imcentras.lt (K.K.); akvile.jarmalaviciute@imcentras.lt (A.J.); justina.pajarskiene@imcentras.lt (J.P.); vytautas.kaseta@imcentras.lt (V.K.); virginijus.tunaitis@imcentras.lt (V.T.); 2 A.I. Virtanen Institute for Molecular Sciences, University of Eastern Finland, 70210 Kuopio, Finland; tarja.malm@uef.fi (T.M.); rashid.giniatullin@uef.fi (R.G.)

**Keywords:** microglia, extracellular vesicles, migration, ATP, P2X4 receptor, MFG-E8, lipid rafts

## Abstract

Extracellular vesicles (EVs) effectively suppress neuroinflammation and induce neuroprotective effects in different disease models. However, the mechanisms by which EVs regulate the neuroinflammatory response of microglia remains largely unexplored. Here, we addressed this issue by testing the action of EVs derived from human exfoliated deciduous teeth stem cells (SHEDs) on immortalized human microglial cells. We found that EVs induced a rapid increase in intracellular Ca^2+^ and promoted significant ATP release in microglial cells after 20 min of treatment. Boyden chamber assays revealed that EVs promoted microglial migration by 20%. Pharmacological inhibition of different subtypes of purinergic receptors demonstrated that EVs activated microglial migration preferentially through the P2X4 receptor (P2X4R) pathway. Proximity ligation and co-immunoprecipitation assays revealed that EVs promote association between milk fat globule-epidermal growth factor-factor VIII (MFG-E8) and P2X4R proteins. Furthermore, pharmacological inhibition of αVβ3/αVβ5 integrin suppressed EV-induced cell migration and formation of lipid rafts in microglia. These results demonstrate that EVs promote microglial motility through P2X4R/MFG-E8-dependent mechanisms. Our findings provide novel insights into the molecular mechanisms through which EVs target human microglia that may be exploited for the development of new therapeutic strategies targeting disease-associated neuroinflammation.

## 1. Introduction

Microglia regulate immune homeostasis in the central nervous system (CNS) by initiating, maintaining, and terminating neuroinflammatory response to all types of injuries [1]. Dysregulated microglial response is important for the development and propagation of various neurological disorders, and therefore the targeting of neuroinflammatory microglia has been considered a promising therapeutic approach [2,3]. Extracellular vesicles (EVs) containing multiple proteins, RNAs, lipids, and metabolites [4] are capable of crossing the blood–brain barrier (BBB) and can be delivered into the brain by the minimally invasive intranasal route [5,6,7]. Intranasal delivery of EVs has been shown to suppress neuroinflammation and promote neuroprotection in different experimental models [8,9,10,11,12]. EVs specifically target and accumulate in pathologically affected areas of the brain [6,13]. Intranasally administered curcumin-encapsulated exosomes are selectively taken up by microglia and suppress secretion of pro-inflammatory cytokines in LPS-treated mice [9]. EVs also reduce microglial activation and prevent the induction of a plethora of pro-inflammatory cytokines after status epilepticus in mice [8]. However, the mechanisms by which EVs regulate microglial responses remain largely unexplored.

During CNS injury, damaged cells release several signaling molecules, including extracellular adenosine triphosphate (eATP) nucleotides that induce recruitment of microglia to the site of the injury and subsequent phagocytosis of apoptotic cells and neuronal debris. ATP potentiates bacterial killing by macrophages through purinergic P2X4 receptors (P2X4R) by increasing the production of mitochondrial reactive oxygen species (ROS) [14]. Blockade of P2X4R signaling inhibits myelin phagocytosis in the experimental autoimmune encephalomyelitis (EAE) model [15]. Purinergic signaling is also crucial for ATP-induced chemotaxis [16,17]. Disruption of local ATP microgradients associated with neuronal hyperactivity during epilepsy impairs microglial motility and phagocytosis [18]. Microglia not only sense eATP by multiple purinergic receptors but can also release ATP by exocytosis [19]. Stimulation of Toll-like receptors (TLRs) triggers Ca^2+^-dependent release of ATP from microglia [19] and macrophages [20]. It has therefore been suggested that controlled ATP release by inflammatory cells may be used to fine-tune autocrine/paracrine responses during acute and chronic inflammation [21]. We have previously reported that EVs derived from stem cells from the dental pulp of human exfoliated deciduous teeth (SHEDs) upregulate human microglia phagocytosis [22]. In the present study, we show that EVs increase intracellular Ca^2+^, trigger ATP release, and significantly enhance microglial motility through milk fat globule-epidermal growth factor-factor VIII (MFG-E8)/P2X4R-dependent mechanisms. Our findings provide novel insights into the molecular mechanisms through which EVs target human microglia.

## 2. Results

### 2.1. Characterization of EVs

**Figure 1:**
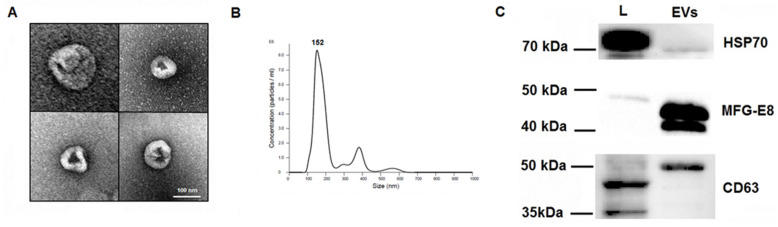
Characterization of extracellular vesicles (EVs) isolated from stem cells from the dental pulp of human exfoliated deciduous teeth (SHEDs). (**A**) Transmission electron microscopy of EVs isolated from SHEDs (120,000x magnification). (**B**) Concentration and particle size of EVs derived from SHEDs as analyzed by nanoparticle tracking analysis using a NanoSight LM10 instrument (Malvern Panalytical). Size distribution of the EVs was around 150 nm. (**C**) Samples from the cell lysates (L) and extracellular vesicles (EVs) were subjected to electrophoresis, blotted, and the membranes probed with antibodies against Hsp70, MFG-E8 and CD63. Bands were visualized by incubation with appropriate horseradish peroxidase-conjugated secondary antibodies and chemiluminescence substrate. Full blots are available in the Supplementary Figures S1–S3.

Transmission electron microscopy (TEM) of EV samples isolated from SHEDs identified vesicles with a typical cup-shaped morphology (Figure 1A); nanoparticle tracking analysis showed that the size distribution of the EVs was around 150 nm (Figure 1B). Western blot analyses revealed that EV fractions were positive for heat shock protein 70 (Hsp70), CD63, and MFG-E8 (Figure 1C).

### 2.2. EVs Increase Intracellular Ca^2+^ Levels in Human Microglia

**Figure 2:**
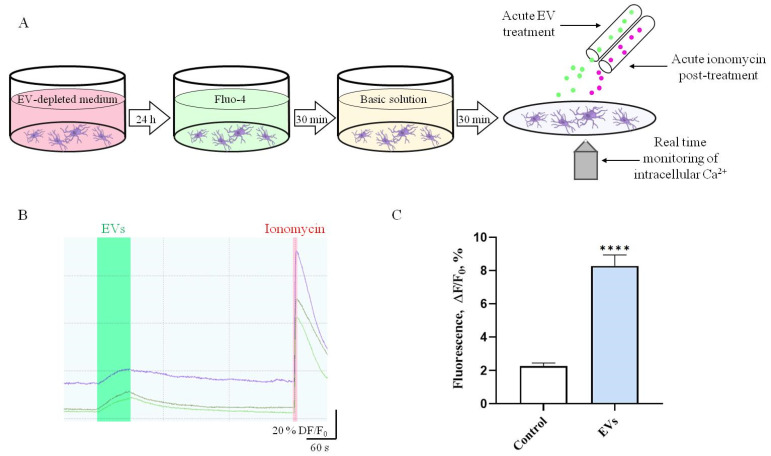
EVs increase intracellular Ca^2+^ and trigger ATP release in human microglial cells. (**A**) Experimental design (please see Materials and Methods section for detailed description). (**B**,**C**) Response of human microglial cells to acute 1 min treatment of EVs. Intracellular Ca^2+^ concentrations in single cells were visualized with the sensitive imaging system (Till Photonics, Kaufbeuren, Germany). The peak of fluorescence in each individual cell during EV treatment was normalized to the peak of fluorescence after response to ionomycin and presented as a percent of intracellular calcium signal. The graph represent mean ± SEM, statistically significant difference was determined by Mann–Whitney test, **** *p* < 0.0001 (Control *n* = 85 cells, EVs; *n* = 127 cells; cells from 3 experiments).

Increases in the mobilization of intracellular Ca^2+^ triggers many functions of microglia including activation, motility, and release of ATP [19,20]. We used live calcium imaging to test how short-term treatment of human microglia with EVs isolated from SHEDs alter microglial intracellular Ca^2+^ levels (Figure 2). Our data show that acute 1 min application with EVs (4 AU/mL) significantly increased Ca^2+^ levels in microglia (by 3.6-fold compared to baseline levels (EV-free BS); *p* < 0.0001, (Figure 2C)).

### 2.3. EVs Trigger ATP Release in Human Microglia

**Figure 3:**
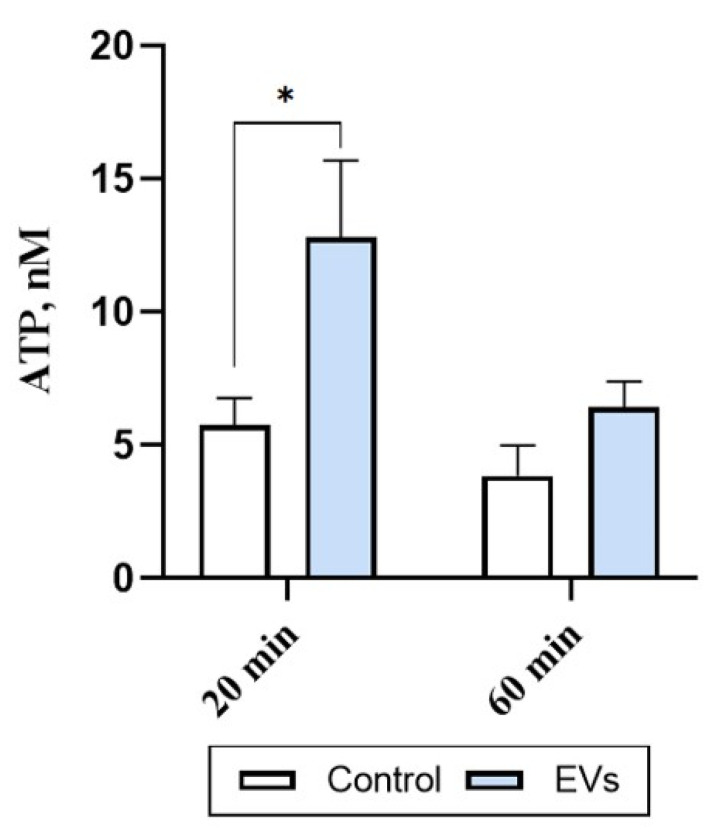
EVs trigger ATP release from human microglial cells. Microglial cells were exposed to EVs (1 AU/well) diluted in BS for 20 and 60 min. Supernatants from each well were subjected to ATP assay. ATP was measured using ATPlite Luminescence Assay System with a Perkin Elmer Wallac 1420 Victor2 instrument using Wallac 1420 software. Each bar represents mean ± SEM, statistically significant difference was determined by two-way ANOVA, Sidak’s multiple comparisons test, * *p* < 0.05 (*n* = 15–20 wells from 3 experiments).

Different biologically relevant stimuli can induce microglial ATP release [19]. We therefore investigated levels of the eATP in human microglia cultures stimulated with EVs (1 AU) for 20 and 60 min. Treatment with EVs for 20 min significantly (by 2-fold; *p* = 0.0158) increased the levels of eATP in the microglia cultures (Figure 3). This increase was no longer evident after 60 min of incubation (*p* = 0.6417) (Figure 3), suggesting that EVs induce a rapid and transient ATP release in human microglia.

### 2.4. EVs Promote P2X4R-Dependent Migration of Human Microglia

**Figure 4:**
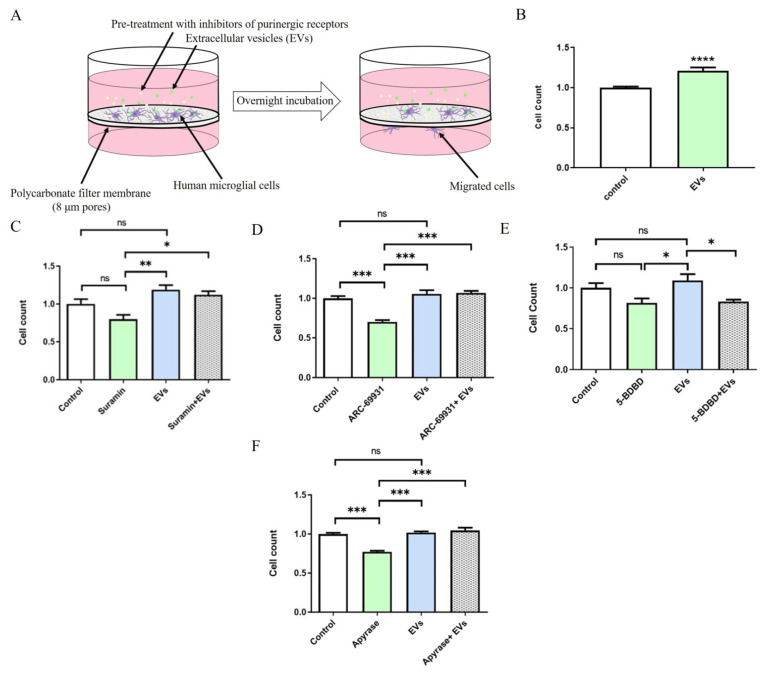
EVs promote migration of microglia through P2X4R-dependent mechanisms. (**A**) Migration assays were performed in a Boyden 10 well chemotaxis chamber (Neuro Probe) using 8 μm pore polycarbonate membranes. Microglial cells were plated into the upper wells, pre-treated for 30 min with inhibitors of different subtypes of purinergic receptors, treated with 1 AU of EVs, and then left overnight. The number of cells that transmigrated to the opposite side of the membrane was determined using optical microscope in three randomly chosen fields from at least three wells for each experimental group. (**B**) EVs promote migration of human microglial cells. Data represent mean ± SEM values, Welch’s test, **** *p* < 0.0001; *n* = 22. (**C**) The effects of nonselective antagonist of ATP-gated P2 receptors suramin (200 µM); (**D**) inhibitor of P2Y12 AR-C69931 (1 µM); (**E**) selective inhibitor of P2X4R 5-BDBD (10 µM); (**F**) ATP degrading enzyme apyrase (20 U/mL). For these experiments, apyrase and 1 AU of EVs were added simultaneously to the upper wells and cells were incubated overnight. Data represent measurements from three independent experiments (only treatment with 5-BDBD from five). The graph represent mean ± SD values, groups were compared with one-way ANOVA, Tukey’s multiple comparisons test, * *p* < 0.05, ** *p* < 0.01, *** *p* < 0.001, ns: not significant.

It is well known that eATP serves as a powerful trigger for motility of microglia [23,24]. We therefore hypothesized that EV-triggered ATP release may promote microglial motility via autocrine and (or) paracrine mechanisms. Indeed, EVs significantly increased microglial migration by 20% (*n* = 22, *p* = 0.0001) in the Boyden chamber assay (Figure 4B). Since microglia are highly sensitive to eATP through several subtypes of purinergic receptors such as P2X4 and P2Y12 [25], we tested different inhibitors to determine which purinergic signaling pathway is responsible for the observed effects. Whilst the nonselective antagonist of ATP-gated P2 receptors suramin failed to significantly alter cellular migration (*n* = 3, *p* = 0.1321; Figure 4C), a highly potent P2Y12 antagonist AR-C69931 significantly decreased migration by 30 percent (*n* = 3, *p* = 0.0009; Figure 4D). AR-C69931 failed to prevent EV-induced migration (*n* = 3, *p* = 0.0002; Figure 4D). These results indicate that suramin and AR-C69931 did not suppress EV-induced migration of microglial cells. P2X4R is relatively insensitive to blockade by suramin; we therefore used the potent selective P2X4R antagonist 5-BDBD [26]. We found 5-BDBD significantly decreased microglial migration in the presence of EVs (*n* = 5, *p* = 0.0263) (Figure 4E). Interestingly, EVs also neutralized the inhibitory effect of the ATP degrading enzyme apyrase on the migration of microglia (*n* = 3, *p* = 0.0001) (Figure 4F). Taken together, our data indicate that EVs activate microglial migration via the P2X4R pathway, and that this effect does not depend on EV-triggered ATP release.

### 2.5. EVs Promote Association between MFG-E8 and P2X4 Receptor Proteins in Human Microglia

**Figure 5:**
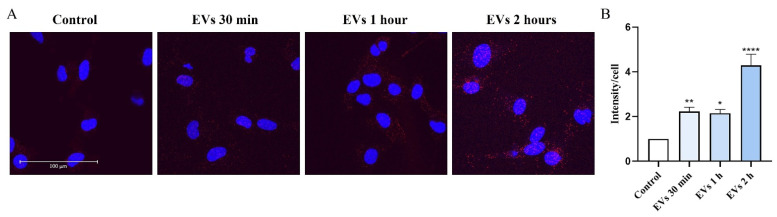
EVs promote association between MFG-E8 and P2X4R proteins in human microglia. (**A**) Confocal images of proximity ligation assay (PLA) dots (red) in human microglial cells. The cells were treated with 1AU of EVs for 30 min, 1 h and 2 h before PLA was conducted. Nuclei, DAPI (blue). Scale bar = 100 μm. (**B**) PLA dots per cell were counted using the ImageJ program. Data shown represent results of 10 fields of view for each experimental group from three independent biological experiments. Data represents mean ± SEM, * *p* < 0.05; ** *p* < 0.01; **** *p* < 0.0001; *n* = 3; Kruskal–Wallis, Dunn‘s multiple comparisons test.

MFG-E8 is a secretory glycoprotein expressed in microglia [27]. MFG-E8 also associates with EVs by binding to the phosphatidylserine (PS) [28,29]. Indeed, EVs derived from SHEDs express high levels of MFG-E8 [22] (Figure 1C). We used a proximity ligation assay (PLA) to detect protein–protein interactions between MFG-E8 and P2X4R in human microglial cells. Our results show an association between MFG-E8 and P2X4R proteins in control (EV-untreated) cells. Exposure to EVs for 2 h significantly (by 4.3-fold compared to control; *p* < 0.0001) promoted association between MFG-E8 and P2X4R proteins (Figure 5), suggesting a close association between MFG-E8 and P2X4R proteins in human microglia which is significantly promoted in cells exposed to EVs.

**Figure 6:**
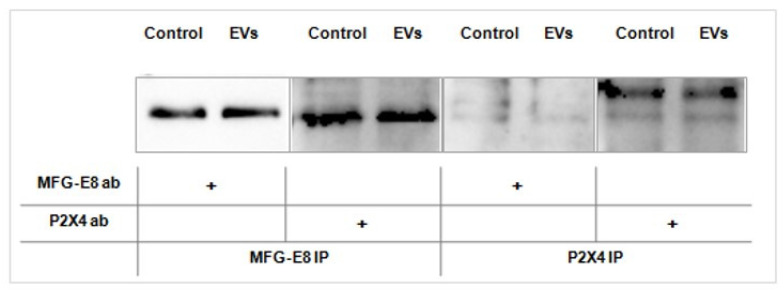
Co-immunoprecipitation of MFG-E8 and P2X4. Representative Western blots showing co-immunoprecipitation of MFG-E8 and P2X4R proteins in human microglial cells treated (or not) with EVs for 2 h, + indicates treatment with appropriate antibody. Full blots are available in Supplementary Figure S4.

Co-immunoprecipitation assays also confirmed the association between MFG-E8 and P2X4R proteins in the microglial cells, but we did not detect increased association after treatment with EVs for 2 h (Figure 6).

### 2.6. Inhibition of MFG-E8 Receptor with Cilengitide Suppressed EV-Induced Migration and Formation of Lipid Rafts in Microglia

**Figure 7:**
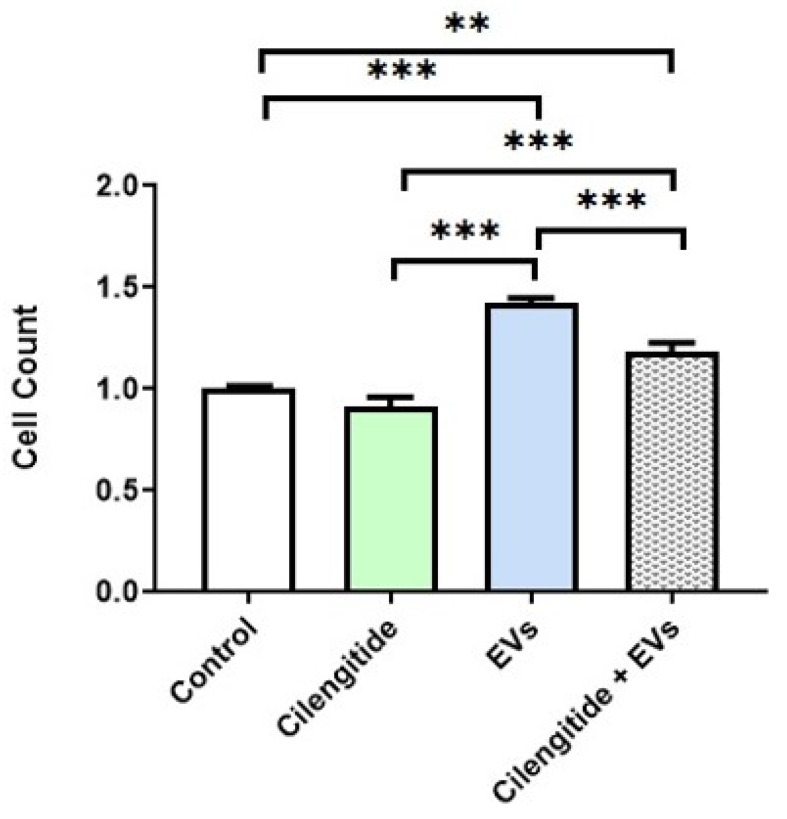
Inhibition of MFG-E8 receptor with cilengitide suppressed EV-induced migration of microglia. For migration experiments (see above) microglial cells were pre-treated for 30 min with inhibitor MFG-E8 receptor cilengitide (10 µM), then treated with 1 AU of EVs. Data represents mean ± SD, ** *p* < 0.01, *** *p* < 0.001; *n* = 3; one-way ANOVA, Tukey’s multiple comparisons test.

MFG-E8 protein interacts with target cells through α_V_β_3_ and α_V_β_5_ integrins [27]. Cilengitide selectively blocks activation of the α_V_β_3_ and α_V_β_5_ integrins and is effectively used as an inhibitor of MFG-E8 signaling [30,31]. We therefore tested the effects of cilengitide on EV-induced migration of microglia. Pretreatment with 10 µM of cilengitide significantly suppressed EV-induced migration of microglia (by 17% compared to control; *n* = 8; *p* = 0.0001) (Figure 7).

**Figure 8:**
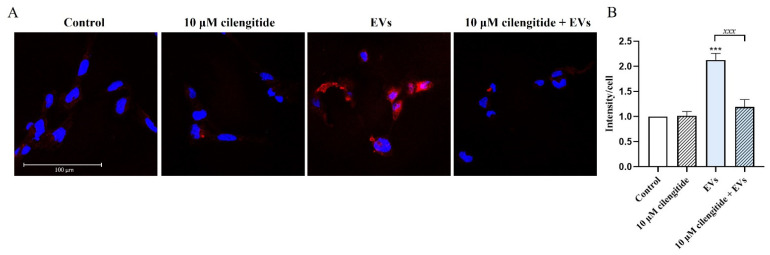
Inhibition of MFG-E8 receptor with cilengitide suppressed EV-induced formation of lipid rafts. (**A**) Confocal images of lipid rafts (red) expressing human microglial cells. The cells were pre-treated with 10 μM cilengitide for 2 h and then treated with EVs (1 AU) of EVs for 30 min before lipid raft labeling and fixation. Lipid rafts were labeled with Vybrant^®^ Alexa Fluor^®^ 594 Lipid Raft Labeling Kit (Thermo Fisher Scientific) according to the manufacturer’s protocol. Nuclei, DAPI (blue). Scale bar = 100 μm. (**B**) The mean fluorescence intensity of lipid rafts per cell were measured with Leica Application Suite X (LAS X) software. Data shown represent the results of 15 fields of view for each experimental group from three independent biological experiments (*n* = 3), plotted as the mean ± SEM. Statistical significance was analyzed by Kruskal–Wallis test using Dunn’s multiple comparison post-hoc test, *** *p* < 0.001; *xxx p* < 0.001.

Lipid rafts serve as organizing platforms enriched with different signaling proteins and receptors initiating inflammatory signaling and different cellular responses [32]. We tested how EVs affect the formation of lipid rafts in microglia. Treatment of human microglia with EVs for 30 min significantly increased lipid raft formation (by 113% compared to control; *n* = 3; *p* = 0.0001) (Figure 8). Again, pretreatment with cilengitide significantly (by 44% compared to control; *n* = 3; *p* = 0.0002) suppressed EV-induced lipid raft formation (Figure 8).

## 3. Discussion

In this study, we demonstrate for the first time that EVs trigger ATP release and promote migration of human microglia through P2X4R/MFG-E8-dependent mechanisms. Our findings provide novel insights into the molecular mechanisms through which EVs target human microglia.

Our initial observation that EVs increased intracellular Ca^2+^ levels and induced rapid ATP release in microglia prompted us to test whether these effects were responsible for increased microglial motility. Blockage of P2X4R with selective inhibitor 5-BDBD prevented EV-induced increases in microglial migration. However, our findings do not support the model according to which EV-triggered ATP release promoted microglial motility via autocrine and (or) paracrine mechanisms. First of all, use of the ATP degrading enzyme apyrase did not suppress the effects of EVs on microglial migration. Furthermore, it is unlikely that EV-induced transient (20 min) increases of eATP could significantly affect overnight migration of cells through the membrane in the Boyden chamber, unless it triggers early remodeling supporting the formation of cell migration mechanisms. Several reports have demonstrated that MFG-E8 secretory glycoprotein is highly expressed in the different types of EVs. By its discoidin domain, MFG-E8 protein associates with PS exposed on the membranes and this property has already been used for the in vivo identification of apoptotic and EV-bound cells [29]. EVs used in our study expressed high levels of MFG-E8 protein (Figure 1C). We therefore tested whether MFG-E8 and P2X4R proteins directly interact in human microglia. Indeed, sensitive in situ PLA revealed a close association between MFG-E8 and P2X4R proteins in EV-treated and untreated cells (Figure 5). Microglial cells constitutively express and secrete MFG-E8 protein [27,33]; it has therefore been impossible to distinguish between microglial and vesicular MFG-E8 fractions. Nevertheless, treatment with EVs remarkably promoted an association between MFG-E8 and P2X4R proteins (Figure 5). To our knowledge, this is the first demonstration of direct interaction between MFG-E8 and P2X4R proteins. The MFG-E8 protein has an epidermal growth factor domain which recognizes integrins α_V_β_3_ and α_V_β_5_ on the target cells [27]. MFG-E8 has been shown to inhibit necrotic cell-induced and ATP-dependent IL-1β production by macrophages through the mediation of integrin β_3_ and P2X7R interactions in primed cells [34]. Several studies have also demonstrated a direct physical association between P2X4R and P2X7R in different types of cells [35,36]. We therefore propose that EV-associated MFG-E8 may interact with P2X4R in human microglia. This statement is further supported by our finding that cilengitide, which is a cyclized RGD-containing pentapeptide that selectively blocks activation of the αvβ_3_ and αvβ_5_ integrins and is effectively used as inhibitor of MFG-E8 signaling [30,31], suppressed EV-induced migration of microglia (Figure 7). P2X4R signaling is important for phagocytosis during EAE [15]. On the other hand, MFG-E8-mediated phagocytic clearance of apoptotic cells by microglia is crucial for the proper regulation of the neuroinflammatory response in the CNS [27,37]. We have also previously demonstrated that EVs increased phagocytic activity of human microglial cells [22]. Although in this study we did not test the effects of cilengitide on the EV-induced phagocytic activity of microglia, we propose that EVs may also promote a phagocytic response through the MFG-E8-dependent pathway. We also suggest that after therapeutic administration, EVs can be selectively recognized and internalized by microglial cells through MFG-E8/αVβ3/αVβ5-dependent mechanisms. This model may at least partially explain selective EV targeting to pathologically affected areas [6,13], and specific accumulation in microglial cells [8,9,10,11,12]. Further studies, especially using MFG-E8-deficient animals are needed to clarify these issues.

Different stimuli promote formation and enlargement of lipid rafts serving as organizing platforms initiating inflammatory signaling and different cellular responses [32]. Lipid rafts are enriched in the innate immune receptors TLRs 2 and 4, TREM2, IFNγR, purinergic receptors P2X and P2Y, integrins and other signaling proteins [32,38,39]. We therefore reasoned that EVs may also affect lipid raft formation in target cells. Indeed, exposure to EVs for 30 min greatly increased lipid raft formation in microglia (Figure 8). Furthermore, pretreatment with cilengitide prevented EV-induced lipid raft formation, showing that EVs trigger lipid raft formation through MFG-E8/αVβ3/αVβ5-dependent mechanisms.

**Figure 9:**
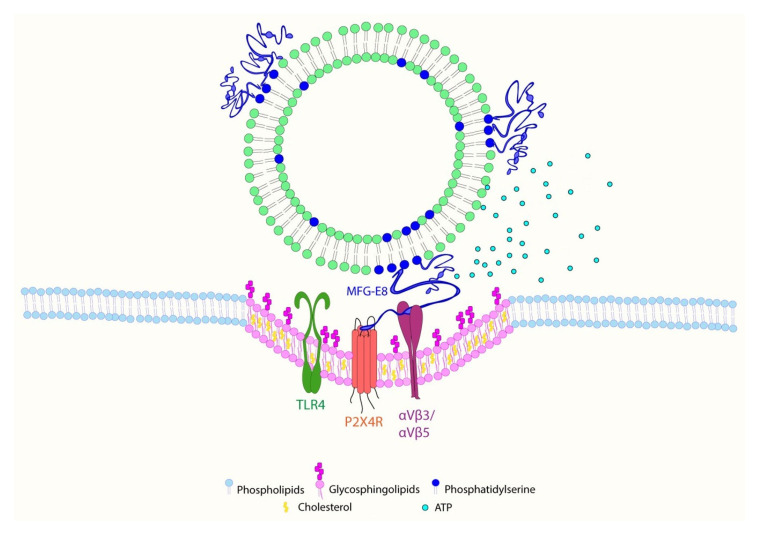
Proposed mechanism for EV action on microglial cells. EVs carrying MFG-E8 proteins associated with phosphatidylserine exposed on the outer membrane are recognized by the αVβ3/αVβ5 integrin receptors of microglial cells and trigger lipid raft formation, interaction with P2X4 receptors, and possibly other molecules enriched in the lipid rafts such as components of the TLR4 multireceptor complex. These events lead to the upregulation of intracellular Ca^2+^, release of ATP, and increased motility of microglia.

Based on these findings, we propose a novel mechanism for EV action on microglial cells (Figure 9).

EVs carrying MFG-E8 proteins are recognized by the αVβ3/αVβ5 integrin receptors of microglial cells and trigger lipid raft formation, interaction with P2X4 receptors, and possibly other molecules enriched in the lipid rafts, such as components of the TLR4 multireceptor complex. These events lead to the upregulation of intracellular Ca^2+^, release of ATP, and increased motility of microglia.

In conclusion, our study demonstrates the importance of the MFG-E8/P2X4 signaling pathway for the immunomodulatory action of EVs in human microglia. Our findings could be potentially exploited for the development of new therapeutic strategies targeting neuroinflammatory microglia.

## 4. Materials and Methods

### 4.1. Culture of Stem Cells from the Dental Pulp of Human Exfoliated Deciduous Teeth (SHEDs) and Human Microglial Cells

SHEDs were isolated according previously described protocol [22]. For the isolation of extracellular vesicles (EVs), SHEDs from the 3rd–5th passages were grown until the cultures reached subconfluence, then standard medium was changed to the serum-free medium MSC NutriStem XF (Biological Industries, Kibbutz Beit Haemek, Israel). Immortalized (SV40) human microglial cell line was purchased from ABM. Human microglial cells were cultivated on cell culture tissue flasks coated with 50 μg/mL of rat tail collagen I (Gibco, Thermo Fisher Scientific, Rochester, NY, USA) in high glucose (4.5 mg/mL) DMEM supplemented with glutamax (Gibco) and 10% of EV-depleted FBS (Biochrom, Berlin, Germany).

### 4.2. Isolation and Characterization of Extracellular Vesicles

Isolation of extracellular vesicles (EVs) was performed using differential centrifugation according to the described protocol [40], with some modifications. All centrifugation steps were performed at 4 °C. Supernatants collected from SHEDs cultivated in serum-free medium MSC NutriStem XF (Biological Industries) were centrifuged successively at increasing speeds (300× *g* for 10 min, 2000× *g* for 10 min, then at 20,000× *g* for 30 min). The final supernatants were ultracentrifuged at 100,000× *g* for 70 min in Sorvall LYNX 6000 ultracentrifuge, with rotor T29-8x50 in oak ridge centrifuge tubes with sealing caps (all from Thermo Fisher Scientific), then the pellets were washed in 40 mL PBS and ultracentrifuged again at 100,000× *g* for 70 min. Final pellets of EVs (exosomal fraction) were resuspended in sterile PBS and stored at −20 °C.

Nanoparticle tracking analysis (NTA) was performed with NanoSight LM10 instrument (Malvern Panalytical, Malvern, UK).

Transmission electron microscopy (TEM) of EVs have been performed according to the previously published protocol [40] with some modifications. First, EVs in PBS were fixed with 2% PFA on ice for 40 min. Then, a Formvar-carbon-coated copper grid was floated on a drop of fixed EV suspension for 20 min at room temperature. Thereafter, the grid was washed in a drop of PBS for 2 min at room temperature. After washing, the grid was incubated in a drop of 1% glutaraldehyde for 5 min at room temperature. Then, the sample was washed 8 times for 2 min by transferring grid from one drop of distilled water to another. The sample was contrasted on a drop of 2% neutral uranyl acetate for 5 min at room temperature in the dark. Afterward, the grid was incubated on a drop of 2% methylcellulose/0.4% uranyl acetate for 10 min on ice in the dark. In the end, the grid was taken by stainless steel loop and air-dried for 5 min. All incubations were performed on a Parafilm sheet with the coated side of grid facing the drop. The prepared EV samples were analyzed with the TEM JEOL JEM-2100F High Resolution EM-20023 (JEOL, Freising, Germany) at 80 kV. The images were taken with an Olympus Quemesa camera, using the iTEM 5.2 software (Olympus Soft-imaging-Systems, Münster, Germany) (Figure 1A).

The yield of EVs prepared from the supernatants of SHEDs grown until subconfluence on the cell culture flasks (representing surface area 37.5 cm^2^) and conditioned for 72 h in serum-free medium MSC NutriStem XF was defined as 1 activity unit (AU). According to the NTA measurements (Figure 1B), 1 AU corresponded to 3.65 × 10^8^ EVs for migration assays and 5.2 × 10^8^ EVs for Ca^2+^-imaging and ATP assays.

### 4.3. Measurements of Intracellular Ca^2+^ Concentration

Human microglial cells were plated on rat tail collagen type I pre-coated coverslips (4000 cells/cm^2^) for 24 h in complete DMEM medium (with 10% FBS and 1% p/S) depleted of EVs by ultracentrifugation at 100,000× *g* for 6 h at 4 °C. Medium was removed and coverslips were loaded with fresh medium containing 10 μM fluo-4 AM for 30 min at 37 °C. After removal of Fluo-4 AM solution, coverslips were moved to basic solution (BS) containing in mM: 152 NaCl, 2.5 KCl, 10 HEPES, 10 glucose, 1 MgCl_2_ and 2 CaCl_2_ for 10 min at 37 °C. Then, cells were placed in the experimental chamber and continuously perfused with BS. Thereafter, changes in the intracellular Ca^2+^ concentration in single cells were visualized with the sensitive imaging system (Till Photonics, Greifelfinger, Germany). Cells were illuminated with the excitation light (480 nm) and the emission light passed through the FITC filter (Chroma Tech., Atlanta, GA, USA). Drugs and EVs were applied by PC-controlled fast application system (Biologic, France) according to the following order: BS for 2 min, EVs (4 AU/mL) for 1 min, BS for 5 min, 10 μM ionomycin for 2 s, BS for 2 min. The peak of fluorescence in each individual cell during EV treatment was normalized to the peak of fluorescence after response to ionomycin and presented as a percent of intracellular calcium signal.

### 4.4. ATP Assay

Human microglial cells were plated in rat tail collagen type I pre-coated 24-well plate (20,000 cells/well) for 72 h in complete DMEM medium depleted of EVs. Medium was removed, cells washed once with BS, then cells were exposed to EVs (1 AU/well) diluted in BS (200 μL/well) containing 100 μM NTPDase inhibitor ARL67156 for 20 and 60 min. Supernatants from each well were collected, heated for 5 min at 65 °C, and subjected to ATP assay. ATP was measured using the ATPlite Luminescence Assay System according to the manufacturer’s instructions with minimal modifications: supernatants were loaded into a flat-bottom white 96-well plate (100 μL/well), then substrate solution was added to supernatants (50 μL/well), and the plate was incubated for 10 min at room temperature on an orbital shaker in the dark. Immediately after incubation, luminescence was measured with a Perkin Elmer Wallac 1420 Victor2 instrument using Wallac 1420 software.

### 4.5. Migration Assay

Migration assays were performed in the Boyden 10 well chemotaxis chamber (Neuro Probe, Gaithersburg, MD, USA), according to the manufacturer’s instructions. Briefly, complete DMEM medium (supplemented with EV-depleted 10% FBS and 1% p/S) was added to the lower wells and separated from the upper wells by a polycarbonate filter membrane with 8 µm size pores. Microglial cells were plated into the upper wells (30,000 cells in 285 μL of complete medium per well), left overnight, and then pre-treated for 30 min with one of the following inhibitors: 200 µM suramin, 1 µM AR-C66931, 10 µM 5-BDBD (all from Tocris, Bristol, UK), or with 10 µM of cilengitide (Sigma, Burlington, MA, USA) for 2 h. Afterwards, cells were treated with 1 AU of EVs and then left overnight. For the apyrase treatments, 20 U/mL apyrase (Sigma) and 1 AU of EVs were added simultaneously to the upper wells and cells incubated overnight. After incubation, membranes were washed 3 times with PBS, fixed with methanol for 5 min, air-dried, stained with 0.25% Cristal Violet for 15 min, and again washed 3 times with PBS. The number of cells that migrated to the opposite side of the membrane was determined using an optical microscope in three randomly chosen fields from at least three wells for each experimental group.

### 4.6. Co-Immunoprecipitation and Western Blot Analysis

For preparation of total cell lysates, cell monolayers were washed twice with cold PBS, pH 7.3, and lysed in Pierce IP lysis buffer (Thermo Scientific, Waltham, MA, USA) supplemented with 1x Halt protease inhibitor cocktail (Thermo Scientific) for 5 min on ice. Samples were centrifuged at 14,000× *g* for 15 min at 4 °C. Supernatants were aliquoted and kept at −20 °C until analyzed. Protein concentrations were measured with the NanoPhotometer Pearl (Implen, Munich, Germany). Lysates were pre-cleared with protein A/G agarose beads (Santa Cruz Biotechnology, Dallas, TX, USA) for 1 h at 4 °C with gentle rocking, centrifuged at 1000× *g* for 30 s and supernatants (200 µg) incubated with either 1 µg of antibody against P2X4 receptor (Alomone Labs, Jerusalem, Israel; APR-002), or 1 µg of antibody against MFG-E8 (Santa Cruz Biotechnology; sc-217574) for 1 h at 4 °C in a rotating device. Then, 20 µL of 50% A/G agarose bead slurry was added into the each vial and incubated at 4 °C overnight in the rotating device. Immunoprecipitates were collected by centrifugation at 1000× *g* for 30 s and supernatants washed 3 times with 1 mL Pierce IP lysis buffer. After final wash pellets were resuspended in the Laemmli sample buffer, heated for 5 min at 95 °C and loaded on Mini-PROTEAN TGX precast gels (Bio-Rad) for electrophoresis in Mini-PROTEAN Tetra cell apparatus (Bio-Rad, Hercules, CA, USA). Gels were then blotted onto a PVDF membrane in a semi-dry Trans-Blot Turbo transfer system (Bio-Rad) and blocked for 1 h at room temperature with 5% BSA (Applichem, Hessen, Germany) in PBS containing 0.18% Tween-20 (PBS-Tw). The membranes were then probed with primary antibodies against MFG-E8 and P2X4 receptor for 1 h at room temperature. After incubation with primary antibodies, membranes were washed three times in PBS-Tw. After washing, membranes were incubated further with horseradish peroxidase (HRP) conjugated secondary antibody (Thermo Scientific) for 1 h at room temperature. The washing procedure was repeated and immunoreactive bands were detected with Clarity ECL Western blotting substrate (Bio-Rad) using ChemiDoc MP system (Bio-Rad).

### 4.7. Proximity Ligation Assay

To detect protein–protein interactions between MFG-E8 and P2X4 in the human microglial cells, a proximity ligation assay (PLA) was conducted using the Duolink^®^ in situ detection kit (Sigma) according to the manufacturer’s instructions. Microglial cells were treated with 1 AU of EVs for 30 min, 1 h and 2 h, then washed with PBS and fixed with 4% PFA for 20 min at RT. After washing three times with PBS, cells were permeabilized with 0.1% Triton X-100 for 15 min, washed three times with PBS again, and blocked with Duolink blocking solution for 1 h at 37 °C. Cells were then incubated with a pair of primary antibodies against MFG-E8 (Santa Cruz Biotechnology; sc-217574) and P2X4 (Alomone Labs; APR-002) (dilution 1:500) for 1 h at RT, washed two times for 5 min with wash buffer A, and incubated with a mix of PLA anti-rabbit PLUS and PLA anti-mouse MINUS oligonucleotide-conjugated secondary antibodies for 1 h at 37 °C. After ligation, amplification with polymerase, and final wash in buffer B, coverslips were mounted onto the slides using Duolink^®^ PLA Mounting Medium with DAPI and analyzed with a Leica TCS SP8 confocal microscope (Leica Microsystems, Mannheim, Germany). Images were taken using a 63x oil immersion lens.

Quantification was performed with the ImageJ program by counting numbers of PLA dots in each cell. The final result was evaluated by dividing the number of PLA dots by the number of cells (total number of cells in each microscope’s field of view was determined by counting of DAPI-stained nuclei). Data were collected in two biological replicates and quantification of PLA dots between P2X4 and MFG-E8 proteins was performed in 8 independent fields of view for each experimental group.

### 4.8. Inhibition of MFG-E8 Receptor

Human microglial cells were plated on rat tail collagen type I pre-coated 24-well plates (for ELISA assays) and on the glass coverslips (20,000 cells/well) for 72 h in complete DMEM medium depleted of EVs (for lipid raft labeling). Microglial cells were pre-treated with 10 µM of cilengitide (Sigma) for 2 h and exposed to EVs (1 AU) for 30 min, then lipid raft labeling was performed.

### 4.9. Assessement of Lipid Raft Formation

Lipid rafts were labeled in live cells using Vybrant^®^ Alexa Fluor^®^ 594 Lipid Raft Labeling Kit (Thermo Fisher Scientific, V-34405) according to the manufacturer’s protocol with minimal modifications. Labeling is based on the specific interaction between fluorescent-labeled cholera toxin subunit B (CT-B) and ganglioside M1 (GM1), which is abundantly expressed in the lipid rafts. After all treatments, cells were washed once with complete growth medium, then incubated with 1 µg/mL fluorescent CT-B for 10 min at room temperature. After washing 3 times with PBS, fluorescent CT-B-labeled lipid rafts were cross-linked with anti-CT-B antibody (200-fold dilution in complete growth medium) for 15 min at room temperature. Thereafter, cells were washed 3 times with PBS and fixed in freshly prepared 4% PFA for 20 min at room temperature. Cells were washed 3 times with PBS and coverslips mounted onto the slides using Duolink^®^ PLA Mounting Medium with DAPI and analyzed with Leica TCS SP8 confocal microscope (Leica Microsystems, Mannheim, Germany). Images were taken using a 63× oil immersion lens.

Quantification was performed with Leica Application Suite X (LAS X) software (Leica Microsystems). The mean fluorescence intensity values taken from each field of view were divided by the number of cells detected (determined by counting DAPI-stained nuclei). Data were collected from two biological replicates, and quantification performed in 10 fields of view for each experimental group.

### 4.10. Statistical Analysis

Statistical analysis was performed from data of at least three independent biological experiments. Graphs represent mean and standard deviation (SD) or standard error of the mean (SEM) values. Differences between 2 groups were compared by Student’s *t*-test, while differences between three or more groups were compared by one-way ANOVA. Data which did not pass the Shapiro–Wilk test of normality were analyzed with non-parametric one-way ANOVA (Kruskal–Wallis one-way analysis of variance) using Dunn’s multiple comparison post-hoc test. All results were considered as significant when *p* < 0.05. Data analyzed using Graph Pad Prism^®^ software version 8.0.1 (Graph Pad Software, Inc., San Diego, CA, USA).

## Data Availability

Data is contained within the article or Supplementary Materials.

## Supplementary Materials

Supplementary Materials can be found at www.mdpi.com/article/10.3390/ijms222010970/s1.
