# Supplementary figures and images for: Extracellular Vesicles from Human Teeth Stem Cells Trigger ATP Release and Promote Migration of Human Microglia through P2X4 Receptor/MFG-E8-Dependent Mechanisms
Source: Int J Mol Sci. 2021 Oct 11;22(20):10970. doi: 10.3390/ijms222010970 (PMC8537493; doi:10.3390/ijms222010970)

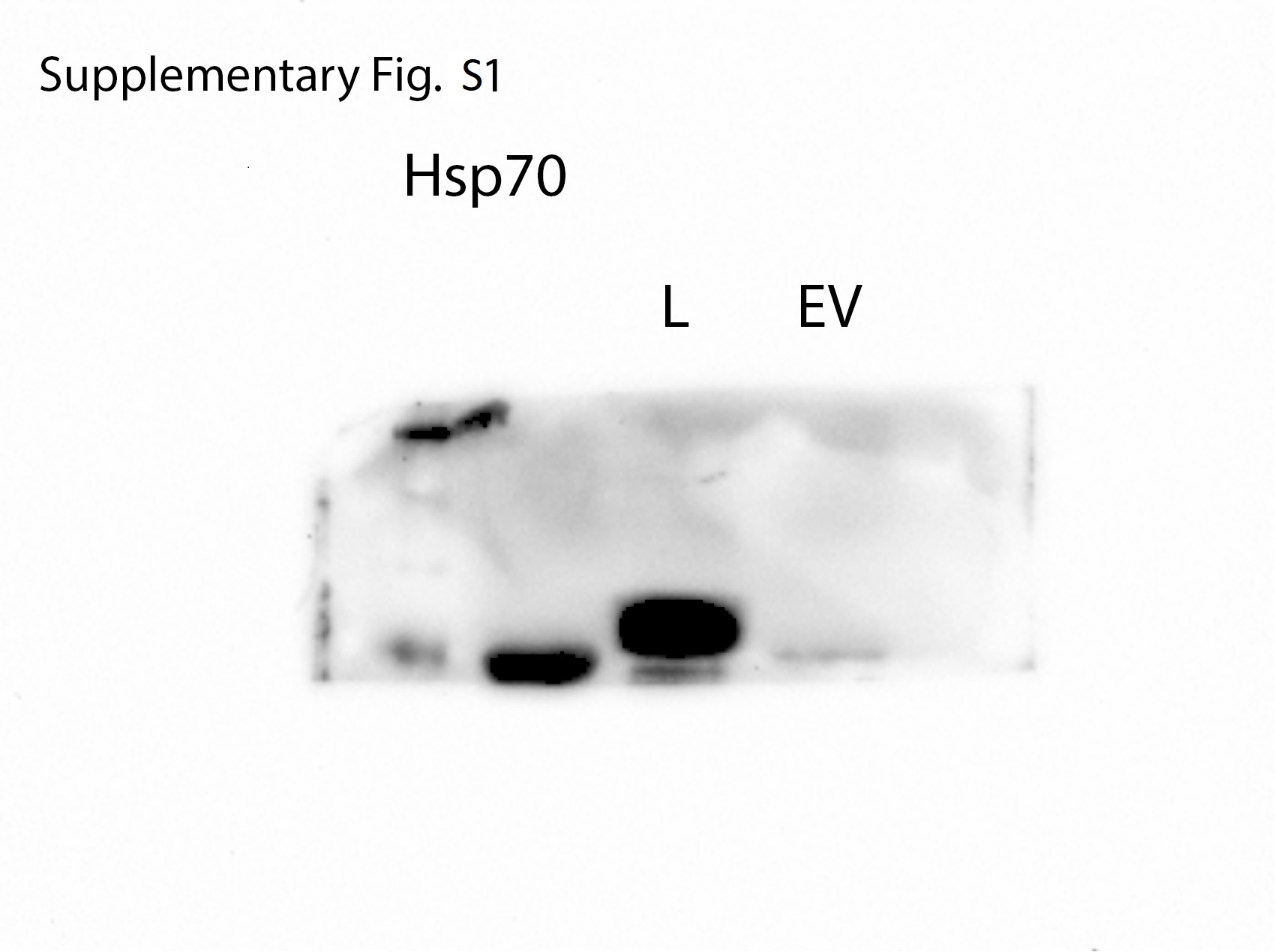

Supplement: Supplementary file 1 [file ijms-22-10970-s001.zip › Supplementary Figure S1.jpg]

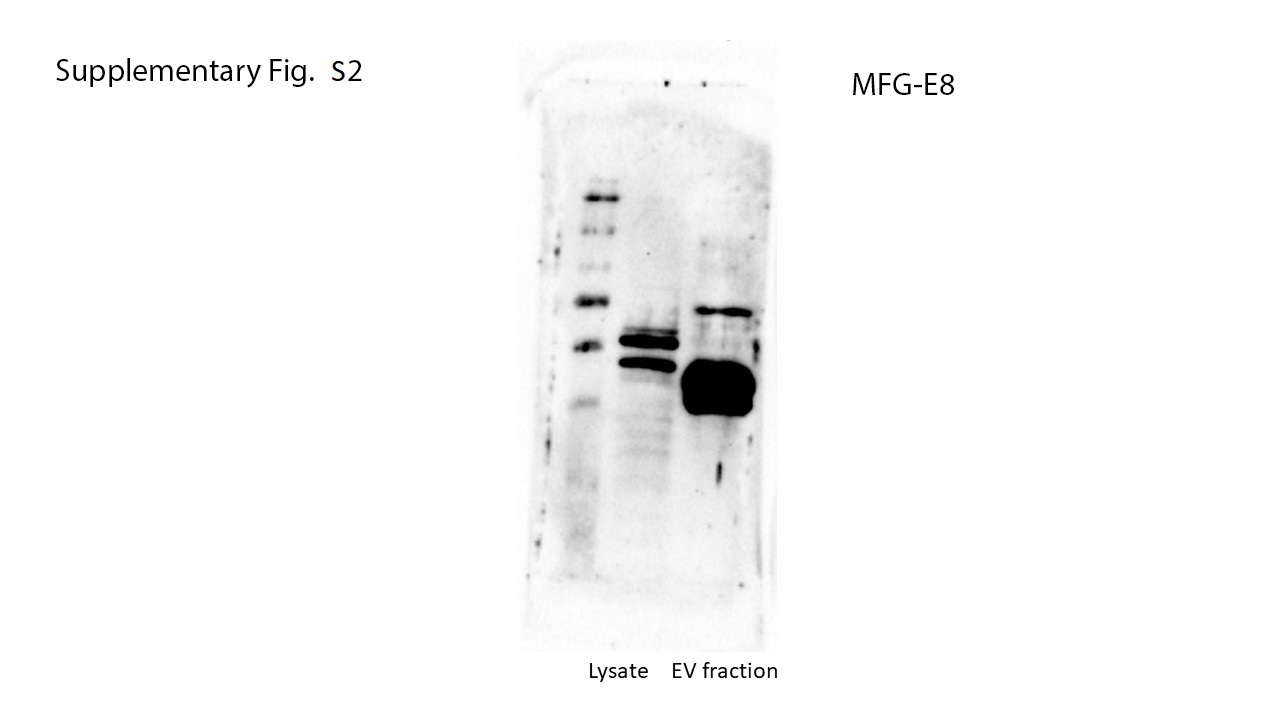

Supplement: Supplementary file 1 [file ijms-22-10970-s001.zip › Supplementary Figure S2.JPG]

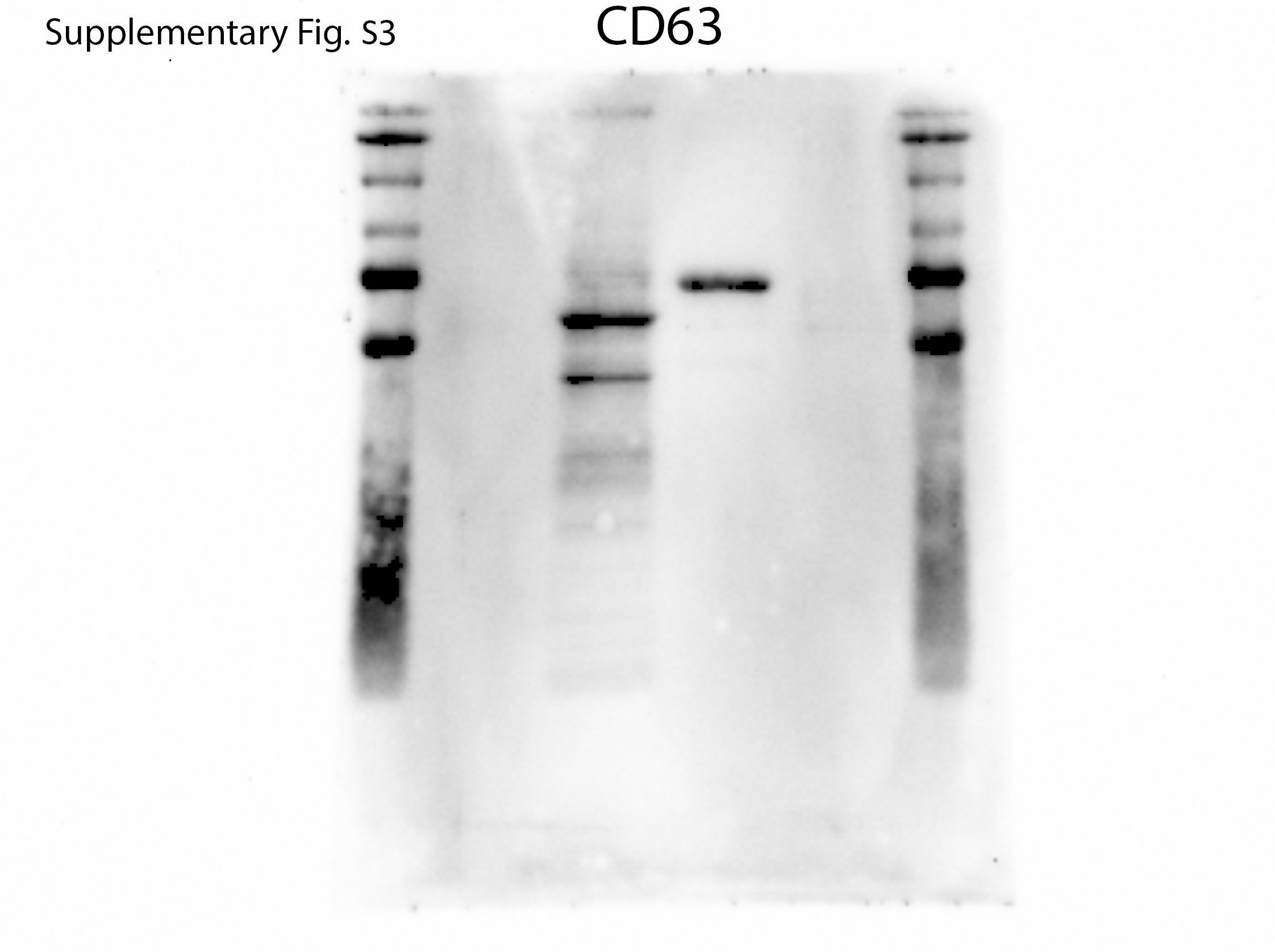

Supplement: Supplementary file 1 [file ijms-22-10970-s001.zip › Supplementary Figure S3.jpg]

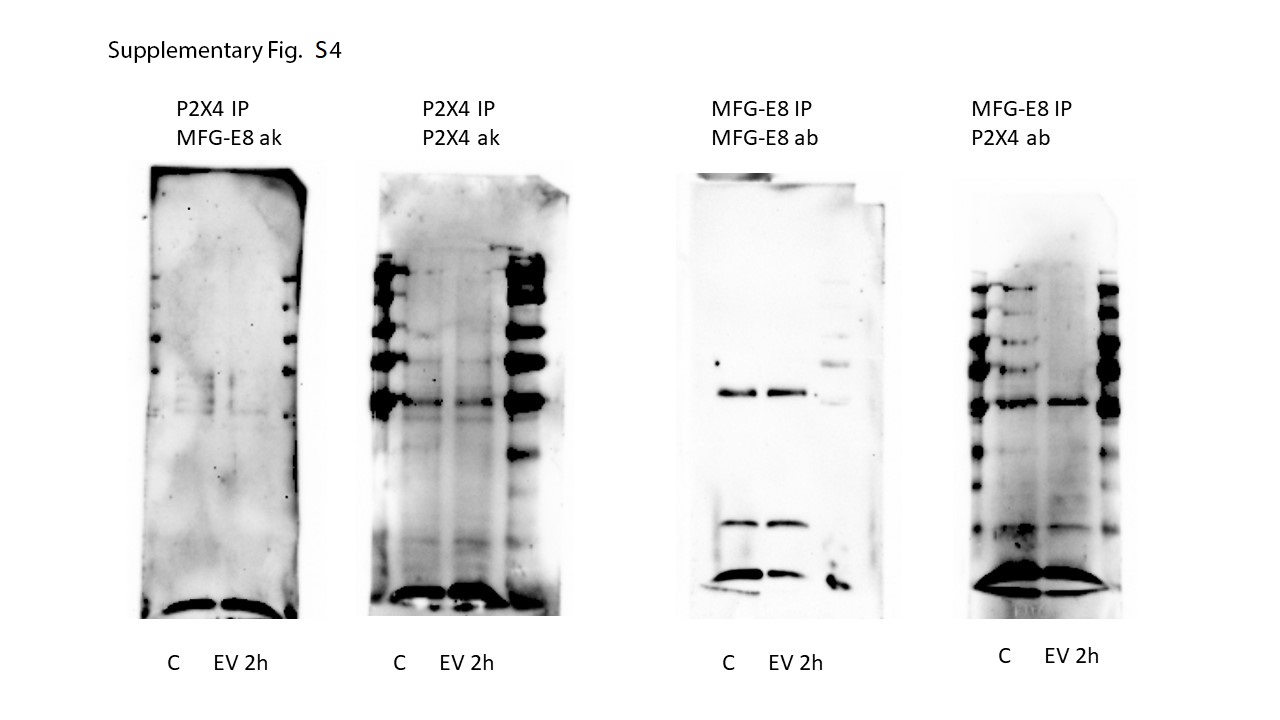

Supplement: Supplementary file 1 [file ijms-22-10970-s001.zip › Supplementary Figure S4.JPG]
